# Antibiotic overuse for acute respiratory tract infections in Sri Lanka: a qualitative study of outpatients and their physicians

**DOI:** 10.1186/s12875-017-0619-z

**Published:** 2017-03-16

**Authors:** L. Gayani Tillekeratne, Champica K. Bodinayake, Thushani Dabrera, Ajith Nagahawatte, Wasantha Kodikara Arachchi, Anoji Sooriyaarachchi, Kearsley Stewart, Melissa Watt, Truls Østbye, Christopher W. Woods

**Affiliations:** 1 Department of Medicine, Duke University School of Medicine and Duke Global Health Institute, Durham, USA; 2 Faculty of Medicine, University of Ruhuna, Galle, Sri Lanka; 3 Nutrition and Indigenous Medicine, Ministry of Health, Colombo, Sri Lanka; 4 Teaching Hospital Karapitiya, Galle, Sri Lanka; 5 Duke Global Health Institute, Durham, USA; 6 Department of Community and Family Medicine, Duke University School of Medicine and Duke Global Health Institute, Durham, USA

**Keywords:** Respiratory tract infections, Antibiotics, Outpatients, Health care utilization, Sri Lanka

## Abstract

**Background:**

Acute respiratory tract infections (ARTIs) are a common reason for antibiotic overuse worldwide. We previously showed that over 80% of outpatients presenting to a tertiary care hospital in Sri Lanka with influenza-like illness received antibiotic prescriptions, although almost half were later confirmed to have influenza. The purpose of this qualitative study was to assess Sri Lankan patients’ and physicians’ attitudes towards ARTI diagnosis and treatment.

**Methods:**

Semi-structured interviews were conducted with 50 outpatients with ARTIs and five physicians in the Outpatient Department (OPD) at a large, public tertiary care hospital in southern Sri Lanka. Interviews were audio-recorded, transcribed, and analyzed for themes related to ARTI diagnosis and treatment.

**Results:**

Patients frequently sought ARTI care in the public sector due to the receipt of free care and the perception that government hospitals carried a sense of responsibility for patients’ health. Patients reported multiple medical visits for their illnesses of short duration and many indicated that they were seeking care in the OPD while at the hospital for another reason. While patients generally expected to receive medication prescriptions at their visit, most patients were not specifically seeking an antibiotic prescription. However, more than 70% of patients received antibiotic prescriptions at their OPD visit. Physicians incorrectly perceived that patients desired antibiotics or “capsules,” a common formulation of antibiotics dispensed in this outpatient setting, and cited patient demand as an important cause of antibiotic overuse. Physicians also indicated that high patient volume and fear of bacterial superinfection drove antibiotic overuse.

**Conclusions:**

Patients in this study were seeking medication prescriptions for their ARTIs, but physicians incorrectly perceived that antibiotic prescriptions were desired. High patient volume and fear of bacterial superinfection were also important factors in antibiotic overuse. Training of physicians regarding guideline-concordant management and dealing with diagnostic uncertainty, education of patients regarding ARTI etiology and management, and systematic changes in the public outpatient care structure may help decrease unnecessary antibiotic prescriptions for ARTIs in this setting.

## Background

Acute respiratory tract infections (ARTIs) are a common reason for seeking ambulatory care worldwide [1]. Although the majority of ARTIs are caused by viruses such as human rhinovirus, respiratory syncytial virus, and influenza, antibiotic overuse for ARTIs is common [2, 3]. In the United States, it is estimated that up to 50% of antibiotics prescribed for ARTIs in the outpatient setting are unnecessary [4]. The overuse of antibiotics is associated with unnecessary adverse drug effects and increased healthcare costs, and is tied to the growing global crisis of antibiotic resistance [5, 6].

In lower-income settings, the problem of antibiotic over-prescription for ARTIs appears to be even worse than in higher-income settings. In Turkey, 100% of children presenting to an emergency room with influenza-like illness (ILI) were prescribed an antibiotic [7]. In rural Thailand, over 80% of outpatients with ILI received an antibiotic prescription [3]. In the Outpatient Department (OPD) of a public, tertiary care hospital in Sri Lanka, we previously showed that 81% of outpatients presenting with ILI received a prescription for an antibiotic, although almost half of these patients were later confirmed by testing to have influenza [8]. Providing physicians with access to rapid influenza test results was associated with a significant decrease in antibiotic prescriptions, but even with results available, 61% of influenza-positive patients received antibiotic prescriptions [9].

Many reasons for antibiotic over-prescription have been described: patients’ lack of knowledge regarding ARTI etiology and demand for a “quick fix,” physicians’ desire for improved outcomes and patient satisfaction, and diagnostic uncertainty regarding the etiology of an ARTI [10, 11]. To improve rational antibiotic prescribing for ARTIs, multi-faceted strategies that target healthcare providers, patients, and the public are recommended, but evidence for the rationale and effect of such interventions has largely been from higher-income settings [6].

To help with future development of ARTI interventions to decrease unnecessary antibiotic use, we assessed Sri Lankan patients’ and physicians’ knowledge and attitudes towards ARTI diagnosis and treatment. We conducted a qualitative study of outpatients with ARTIs and their OPD physicians at the same tertiary care hospital as our prior ARTI studies [8, 9].

## Methods

### Setting and participants

The interviews for this study were conducted from July- August 2015 at a large public, tertiary care hospital in southern Sri Lanka. This hospital serves as a referral center for outpatient and inpatient care, although the majority of care is for patients who live in surrounding areas. A total of 25 pediatric patients and 25 adult patients were recruited for enrollment. Consecutive patients meeting World Health Organization criteria for influenza-like illness (fever and cough in the prior 7 days with no other etiology) were approached for enrollment by a MBBS-qualified local study physician [12]. Self-reported fever was used to determine eligibility. Consent was obtained from patients ≥18 years of age and from the guardians of patients 1–17 years, and assent was obtained from patients 12–17 years. For pediatric patients, the interviews were conducted with the patients’ guardians. All interviews were conducted after patients had received care from the OPD physicians.

All physicians working in the OPD of the teaching hospital were also approached for enrollment. The OPD serves >1000 patients daily between the hours of 8 am and 7 pm. The OPD provides primary care to patients presenting with acute and chronic complaints. There are three shifts of physicians working each day in the OPD. From a total of 13 OPD physicians, five agreed to participate in the semi-structured interviews. An informational session was conducted with all OPD physicians as a recruitment tool. Prior study results regarding influenza prevalence and antibiotic prescription frequencies were discussed and the rationale for the current qualitative study was explained [8, 9]. The informational session was conducted after two physician interviews had been completed.

### Procedures

The patient and physician interview guides were developed by the study team, which consisted of both Sri Lankan and American physician-researchers. The study team also included two investigators with expertise in qualitative research.

The patients’ interview guide covered three domains:

1. Care-seeking patterns for ARTIs
2. Knowledge of ARTI etiology and treatment
3. Assessment of treatment received at the OPD visit

The physicians’ interview guide covered four related domains:

1. Approach to the diagnosis and management of ARTIs
2. Physicians’ reasons for potential antibiotic over-prescription for ARTIs
3. Physicians’ understanding of antibiotic resistance and impact of resistance on antibiotic prescribing patterns
4. Opportunities for improving the care of patients with ARTIs in the OPD

Given the straightforward nature of the questions, interview guides were not pretested. Patient interviews were conducted in Sinhala by a local study physician (AS) and lasted approximately 15 min. Physicians’ interviews were conducted in English or Sinhala (according to the choice of the interviewee) by an infectious disease-trained bilingual physician (LGT) and lasted approximately 30 min. Written informed consent was obtained to audio record the interview and to use the content for analysis and publication. The interviewer used open-ended questions from the interview guide and followed up with probes to elicit additional information or to clarify prior responses. Information regarding medications prescribed at the visit was obtained by reviewing prescriptions provided by physicians or inspecting medication packets dispensed by the pharmacy.

### Data analysis

Each interview was audio recorded and transcribed verbatim, and interviews conducted in Sinhala were translated into English. A two-stage coding process was used with structural coding followed by thematic coding [13]. Structural coding followed the content of the interview guide. Thematic coding was based on themes that arose from structural coding. The structural and thematic codes were discussed by members of the study team (LGT, TD, and TO) until consensus was reached regarding differences in interpretation. Coding was primarily deductive in nature, but the study team allowed for any inductive coding of unexpected findings. NVivo (Version 10.2, QSR International) was used to perform initial coding and categorization of relationships.

## Results

### Patients

We enrolled twenty-five adult patients and 25 pediatric patients with ARTIs who were seeking care in the OPD. Among adult patients, the mean age was 49 years (range 18–80) and 56% were female. Among pediatric patients, the mean age was 9 years (range 1–17) and 56% were female. Mean age of the pediatric patients’ caregivers was 42 years (range 27–61) and 92% were female, with 80% of caregivers being the patients’ mothers.

Within the three domains of the patients’ interviews, seven themes emerged and are listed below. Representative quotes within each theme are listed in Table 1. In general, responses of adult patients and the caregivers of pediatric patients were similar and thus were grouped together.

**Table 1:** Domains, themes, and representative quotes for outpatients seeking care for acute respiratory tract infections (ARTIs) at a tertiary care hospital in southern Sri Lanka

Domains	Themes	Representative quotes
Health-seeking behavior	Public sector preferred for ARTI care	*“We don’t have money to take medicine from outside, so whatever the illness we get, we come here….”*- OPD024 *“No special reason, but there are good doctors in government hospitals. We believe that unnecessary drugs are not given from hospitals. When we are taking from dispensaries (private pharmacies), they give unnecessary drugs.”-* OPD042
	Multiple medical visits for ARTIs	*“Today my wife has a clinic for a wound in the leg. Came for that, so took medicine at the same time.”-* OPD021 *“Took [medications for daughter] from the village…. It was a bit better, but as I came to take medicine, at the same time I took medicine for her from here as well.”-* OPD027
Knowledge of ARTIs	Non-microbial etiology for ARTIs	*“*[ARTIs are] *due to heavy rain. N_ is full of mountains, so it is cold there. Other thing is eating foods in the fridge, that also causes wheezing”-* OPD026 *“*[ARTIs are] *due to dust in the surroundings. There are lots of vehicles these days, so usually when I come I cover my nose. I get this illness even when I am exposed to a small amount of dust”-* OPD039 *“He eats ice cream all the time, he is always playing in the water, because of those reasons”-* OPD061
	Medications are needed to treat ARTIs	*“No I will not accept it [if medications are not prescribed]. A mother is taking a child to the hospital as there is a difficulty due to the child’s illness. The mother knows the difficulty of the child, whatever the hospital says. So I will not be satisfied. Usually I bring the child here when the disease is severe…. Then I will go to the private sector”-* OPD037 *“Cannot accept [if medications are not prescribed]. Yesterday night I feel like dying due to severe cough…. Then I will come back another day and take”-* OPD047 *“Then we are disappointed… I will go to T_ Hospital. If I have money, I will go to the private sector. Then I don’t have to wait in the queue as well, it saves time. But we need money for that. Most of the time as we do not have money, we go to government hospitals.”-* OPD031
	Low level of knowledge regarding antibiotics	*“When taking it, disease severity reduces. Also given to cure the wounds”-* OPD036 *“I thought that it is given for phlegm?”-* OPD056
Treatment received during visit	Receipt of multiple prescriptions is common	*“Although I came late, there was a nurse that I know, so could get the medications soon* [from the OPD]*. I can be satisfied as I also got a lot of medications”-* OPD031
	Patients were satisfied with care received	*“Yes [I am satisfied]…. Now I have received medications. I will go home and take these. Hope that I will be well.”-* OPD024 *“Yes [I am satisfied]…. When we take these drugs properly, the illness cures in two days. Our fault is that we do not care of ourselves. When the illness gets better in one day with medications, we bathe, eat whatever we want, that way we make it worse.”*- OPD025

### Domain 1: Health-seeking behavior

#### Public sector preferred for ARTI care

The majority (33, 66%) of patients indicated that they generally sought care for ARTIs in the public sector. Multiple reasons were cited, including the receipt of free care, faith in the public healthcare system, and the belief that the public healthcare sector carried a sense of responsibility for patients and the care delivered: “Not like other places. Here there is a sense of responsibility. Although I take medicines from private places, I again have to come to a government hospital, so it’s better to come here.” Among adults who had previously sought care in the private sector, convenience was one of the key reasons cited, since public hospital OPD wait times were long and interfered with work hours: “It saves time rather than coming to the OPD. If coming to the OPD, then have to spend a whole day for it.”

#### Multiple medical visits for ARTIs

Although all enrolled patients had an active respiratory illness of one week’s duration or less, almost half (24, 48%) of patients indicated that they had sought prior care for the same illness. Several patients indicated that they had made more than one prior medical visit for the same illness: “Yes took previously from that private doctor, but did not cure, then took from N_ Hospital, still it is not cured, so came here.” In addition, although not asked directly, 13 (26%) patients indicated that they were at the hospital for another reason (i.e., a clinic visit), and had decided to visit the OPD at the same time for their ARTI: “I didn’t come for this today. I came to take some medicine for my son. At the same time, took [medicine] for me as well.”

### Domain 2: Knowledge of ARTIs

#### Non-microbial etiology of ARTIs

When asked open-ended questions about the etiology of their illness, the majority (46, 92%) of patients described non-microbial causes such as bathing, drinking cold water, eating cold foods, or getting exposed to rain: “I did not take care of myself, I drank cool water, bathed at night, often I was under the fans.” These reasons were also cited as explanations for the worsening of ARTI symptoms: “It gets worse if you are not careful, if you get wet, if you use cold water.” Other non-microbial causes of ARTIs that were listed included dust exposure, smoking, or drinking alcohol. Only four patients mentioned infectious etiologies such as viruses, bacteria, or germs.

#### Medications are needed to treat ARTIs

Self-treatment prior to the OPD visit was common among patients. Most patients (43, 86%) had tried traditional therapies before their visit to the OPD. Common therapies that were mentioned included drinking Ayurvedic herbal preparations (*n* = 25, 50%), drinking boiled coriander (*n* = 20, 40%), drinking or eating ginger (*n* = 7, 14%), and steam inhalation (*n* = 17, 34%). In addition, 16 (32%) patients had used acetaminophen for their fever prior to presentation.

A total of 18 (36%) patients felt that some form of medication was always necessary for treating ARTIs. Another 21 (42%) felt that treatment with medications was only necessary if the illness were to become worse or severe. However, the majority of patients (*n* = 39, 78%) expected a medication prescription from their visit and more than half (*n* = 28, 56%) would not accept it if the physician said that medications were not needed for their illness: “But I can feel it right…. I can feel that I am seriously sick.” Several patients said that if they were not prescribed medications, then they would seek care from another provider until they received a prescription: “No, I will not accept it [if medications are not prescribed]. A mother takes a child to the hospital because the child is having difficulty due to illness. The mother knows the difficulty of the child, whatever the hospital says. So I will not be satisfied…. Then I will go to the private sector.”

#### Low level of knowledge regarding antibiotics

One third of patients and caregivers (*n* = 16, 32%) did not know of any specific treatments needed for ARTIs. Commonly mentioned medications included acetaminophen (36%), the antihistamine chlorphenamine (15, 30%), amoxicillin (14, 28%), vitamin C (8, 16%), a bronchodilator such as salbutamol (5, 10%), and “capsules” (5, 10%). More than half (*n* = 29, 58%) of patients had heard of antibiotics, but only five (10%) knew that these medications worked on microbes or impacted the immune system-- “I have heard names, I don’t know what they are taken for.” Only five (10%) felt that antibiotics should be used for treatment of all ARTIs. With the exception of two patients who went to pharmacies to ask for antibiotics, no other patients reported going to another healthcare facility or provider to specifically request antibiotics.

### Domain 3: Treatment received during visit

#### Receipt of multiple prescriptions is common

All patients received prescriptions for medications, with most receiving multiple prescriptions. The mean number of unique prescriptions received was 3 (range 1–5). The majority (*n* = 36, 72%) received a prescription for an antibiotic, with most of these (*n* = 27, 75%) being for amoxicillin, followed by 4 (11%) for ciprofloxacin. Other common drugs prescribed included acetaminophen (*n* = 31, 62%), the antihistamine chlorpheniramine (*n* = 24, 48%), salbutamol (*n* = 17, 34%), dexamethasone (*n* = 14, 28%), prednisolone (*n* = 6, 12%), and vitamin C (*n* = 7, 14%). No patients received laboratory testing for diagnostic purposes, and most patients (*n* = 39, 78%) did not expect any diagnostic testing.

#### Patients were satisfied with care received

Although an alarming 94% of patients indicated that the doctor had not explained what medications had been prescribed or the indications for these medications, the majority of patients (*n* = 34, 68%) were satisfied with their visit. None said that they would visit another doctor after the OPD visit to obtain further prescriptions or care, unless their illness did not improve: ““Yes. Because I got the medicine for the illness, I am satisfied.”

#### Physicians

Among physicians, the mean duration of time on staff in the study hospital’s OPD was 3 years (range 0–8), and the mean time since obtaining the last formal medical degree was 21 years (range 8–33). Four out of five physicians reported that they routinely treated as many as 100–150 patients daily in the OPD.

Within the four domains of the physicians’ interviews, nine themes emerged and are listed below. Representative quotes within each theme are listed in Table 2.

**Table 2:** Domains, themes, and representative quotes regarding acute respiratory tract infection (ARTI) diagnosis and treatment for OPD physicians at a tertiary care hospital in southern Sri Lanka

Domains	Themes	Representative quotes
Diagnosis and management of ARTIs	Clinical symptoms can help differentiate viral and bacterial ARTIs	*“At the beginning, same symptoms. After 2–3 days we can differentiate… If continuous fever, and if patients complain more and more, develop more severe illness, are more dyspneic, have more fever, and are more acutely ill [then it is bacterial]”-* OPD003 *“Viral of course, it is mild fever, sometimes body aches, headache, loss of appetite, so on. Based on our own experience, we know…. Viral of course nothing but symptomatic and supportive treatment…. We give paracetamol, piriton [antihistamine], and so on.”-* OPD004
	Laboratory testing in the OPD is minimal	*“It’s a problem because we can’t do cultures here. Blindly we treat with antibiotics usually.”-* OPD002 *“So we do the basic investigations and see for the response and after that we treat…. Yes, if we can get that rapid testing then the treatment will be very vigilant. And we can expedite.”-* OPD004
Antibiotic over-prescription	Patients demand antibiotics	*“That’s tricky because patients here, in Sri Lanka I don’t know, they sometimes ask for antibiotics because they are used to getting them…. They take very low doses of antibiotics. And they come and say this is what I’m taking. So sometimes we have to continue the antibiotics with the normal dose. Like the adult dose…. They say, ‘With antibiotics only, I get relief.’ So it’s a nuisance to us.”* – OPD001 *“Medically not indicated, but we have to weigh the conditions and the patient. Some patients, they are coming while they are going to work…. They are finding day-to-day income. The family gets disturbed due to the lack of income. Most of the time, they are asking for a one-shot treatment…. They say ‘Sir, there is no capsule given here’- that is what. They are experienced, they need some form of capsules.”-* OPD004
	Diagnostic uncertainty and bacterial superinfection	*“Viral ones we sometimes treat with some antibacterial cover-up, otherwise they will develop secondary bacterial infections. Usually we do so.”-* OPD003 *“For viral-- joint pain, back ache, loss of appetite [are common]…. If I’m sure, then I don’t give an antibiotic. If I’m not sure, I often use an antibiotic.”-* OPD005
	Children are more vulnerable than adults	*“Children of course, I start antibiotics a little bit earlier than with adults. Because with my experience, I think that even at home I give them antibiotics a little bit earlier. If my child is ill for 3–4 days, I start antibiotics. Sometimes I practice that in the OPD as well. In my experience, I think that they get better. I don’t know if it’s the wrong thing.”-* OPD001 *“A child needs special attention…. Sometimes on the first or second day we try anti-inflammatories and antibiotics.”-* OPD003
	Competition with private sector/other doctors	*“Competition is if I am not prescribing an antibiotic from the first day, sometimes it can be a bacterial one [infection], and on the second, third day the patient will get more severe symptoms and go to another practitioner. Then [the practitioner will say] ‘this is pneumonia’ and they will start [antibiotics]. Then the first person will get less marks…. Mother or some relative will say that this is the best physician…. His treatment is much better than the other one, then the first person will get less respect. Because of competition, most of the time in the private sector, they use antibiotics.”-* OPD003
Antimicrobial resistance	Lack of awareness of antimicrobial resistance	*“But actually, about antibiotic resistance, so far I have no experience. Just by treating patients very often, we give treatment for three or four days. After that sometimes they won’t come to us. But bit difficult to assess whether they have developed antibiotic resistance or not.”-* OOP004
Opportunities for improving ARTI care	Continued physician trainings	*“What I think is that if you can educate our doctors from time to time, with these epidemics, their nature, and how to treat them, then of course it is really beneficial. I’ll tell you that most of the doctors do not read about these conditions. They won’t go through these leaflets or those results papers.”-* OPD004
	Need for systematic changes	*“If there were a longer time duration in the OPD for the patients. If we can allocate 5 min or so per patient, then we can improve things…. If something is given free of charge, they will take anything. While just waiting, they come and get medications…. The patient who comes to go to the bank gets a ticket from the OPD* [number to wait in line]*, goes to the bank to get their work done, then comes again to get medication. Actually, it’s not for a real need that they take medications…. Not a large amount, but [by charging] a small amount like 50 Rupees, it’s possible to reduce it. If five people come, all five will get a ticket. If you charge 50 Rupees, then only the person in need will take the medication.”-* OPD005

### Domain 1: Diagnosis and management of ARTIs

#### Clinical symptoms can help differentiate bacterial and viral ARTIs

All physicians indicated that they used patients’ clinical presentations to try to differentiate between bacterial and viral illnesses. Viral illnesses were thought to be of shorter duration, milder symptoms, and associated with sick contacts, and were treated symptomatically. Bacterial infections were thought to be of longer duration and associated with more severe symptoms, and were treated with antibiotics: “Yes, if it is more than three days and with fever, we suspect that they’re having bacterial infections and we automatically without checking the blood start antibiotics.” However, one physician indicated that it could be difficult to differentiate between viral and bacterial infections based on symptoms alone: “High fevers, chills, muscle pain, vomiting [are seen with bacterial infections]. They come for both [viral and bacterial infections] though, hard to tell exactly.”

#### Laboratory testing in the OPD is minimal

All physicians indicated that only basic testing capabilities such as full blood counts, blood smears, and erythrocyte sedimentation rate were routinely available through the OPD for diagnosing patients with ARTIs. Two physicians indicated that the available testing was adequate based on the constraints of the OPD setting, including high volume of patients and limited time with each patient: “I mean yes, the tests are important, but from our point of view, for our management, I think that is enough for us.” But two others indicated that more testing would be helpful: “So can’t get a proper diagnosis here…. If we have sputum cultures freely available, it’s better. And chest x-ray facilities.”

### Domain 2: Antibiotic over-prescription

#### Patients demand antibiotics

A consistent theme that emerged from all physicians as a reason for antibiotic over-prescription was that patients asked for, and even demanded, antibiotics. In some instances, patients specifically asked for the antibiotic amoxicillin: “Sometimes they are asking for amoxicillin. They are specifying the name and asking for amoxicillin. They say, ‘earlier I took amoxicillin, and it responded. Can you kindly give?’” Physicians noted that even if patients were not always able to remember the name of a drug, they may demand a “capsule,” which is the common formulation for amoxicillin at this hospital: “Some patients, they are coming while they are going to work…. Most of the time, they are asking for a one-shot treatment…. They say ‘Sir, there is no capsule given here.’ They are experienced. They need some form of capsules.”

#### Diagnostic uncertainty and bacterial superinfection

Four physicians felt that antibiotic overuse may also be driven by diagnostic uncertainty and the fear of missing a bacterial infection or superinfection: “If 100% not sure clinically, an antibiotic is used.” In addition, when it was difficult to obtain details regarding the illness, physicians often prescribed an antibiotic: “When it’s rushed, it’s difficult to get a lot of details. And there are also patients who are not cooperative. In those instances, we give an antibiotic.”

#### Children are more vulnerable than adults

Three participants felt that children were more vulnerable with their illness than adults, and thus were more likely to be started on antibiotics: “Children of course, I start antibiotics a little bit earlier than with adults…. If my child is ill for 3–4 days, I start antibiotics. Sometimes I practice that in the OPD as well. In my experience, I think that they get better. I don’t know if it’s the wrong thing.” Children were also more likely to be started on antibiotics because they were less able to communicate their symptoms and were more difficult to examine: “For children…. It’s difficult to explain symptoms…. They cry a lot, are irritable, lung signs are difficult to hear. Because of that, a lot of times we use [antibiotics].”

#### Competition with private sector/other doctors

One participant indicated that competition with other physicians drove antibiotic over-prescription in the private sector: “If I am not prescribing an antibiotic from the first day, sometimes it can be a bacterial one [infection]… The patient will get more severe symptoms and go to another practitioner. Then [the practitioner will say] ‘this is pneumonia’ and they will start [antibiotics]…. Mother or some relative will say that this is the best physician…. Because of competition, most of the time in the private sector, they use antibiotics.” This competition could potentially have a bearing on physicians’ actions in the public sector, since many physicians practice in both the public and private sectors. Another participant felt that there was a lack of knowledge among other doctors regarding antibiotic over-prescription, and that if he/she did not prescribe an antibiotic, patients would seek care from another OPD physician: “Yes, yes indiscriminate use of antibiotics…. Unfortunately I have observed doctors prescribe just like parrots…. Reason is lack of knowledge…. If I do not prescribe, he will go to another room and then that doctor would ask ‘you didn’t have worm treatment for a long time?’ when they say abdominal pain, [the doctor] would say ‘then take worm treatment.’”

### Domain 3: Antimicrobial resistance

#### Lack of awareness of antimicrobial resistance

Three physicians felt that there was limited awareness and information regarding antibiotic resistance: “I have heard, but we don’t get much information about resistance.” One participant indicated that fear of antibiotic resistance drove him/her to use broader-spectrum antibiotics for ARTIs: “Because patients are not responding to small antibiotics like amoxicillin…. I don’t usually prescribe amoxicillin because I have realized by myself that it doesn’t work, maybe because of that resistance. So usually I prescribe for an adult ciprofloxacin because it is freely available here. If they can buy from outside, according to the clinical level, I will go to co-amoxicillin.”

### Domain 4: Opportunities for improving ARTI care

#### Continued physician trainings

Most physicians (4) indicated that few or no continued trainings regarding ARTI management were available through the hospital. Four participants indicated that having local access to training or workshops regarding ARTI management would be beneficial: “That kind of material is needed…. Most of the senior physicians sometimes stay in the OPD…. After internship they are not going to work in the ward or something. If we could get some way to refresh their knowledge, since time to time indications and guidelines change.” Two physicians stated that time constraints due to their workload prevented them from attending any sessions that might have been available.

#### Need for systematic changes

Systematic changes were also suggested as potential solutions to improve care. One participant felt that high patient volume affected the quality of care that was delivered: “See if I examine more than 100 patients here, actually there is no quality as such. Somehow we have to do it… We have to clear out the patients…. I am not satisfied with the system, but anyway I have to do that, I am compelled to do so.” Another suggested that the volume of patients was high because patients often presented with minor complaints. One physician suggested that charging a nominal fee might help limit use of the OPD to those patients who actually needed medical care: “Not a large amount, but [by charging] a small amount like 50 Rupees (USD 0.38), it’s possible to reduce it. If five people come, all five will get a ticket. If you charge 50 Rupees (USD 0.38), then only the person in need will take the medication” [14]. The issues of high patient volume affecting quality of care, and of patients seeking care with minor ailments, were echoed by other physicians as well.

## Discussion

In this qualitative study of outpatients and their physicians at a public hospital in Sri Lanka, antibiotic prescriptions for ARTIs were common (70%). Interestingly, there was a disassociation between patients’ expectations for antibiotics and physicians’ perceptions of patient expectations. Patients generally had a low level of knowledge regarding ARTI etiology and antibiotics, with the main expectation for treatment being the receipt of a medication prescription. Physicians incorrectly perceived that patients desired antibiotics, and also cited factors such as diagnostic uncertainty, competition with the private sector, and lack of time as reasons for possible antibiotic over-prescription. Patients were generally satisfied with treatment received in the OPD, but physicians identified both systematic and patient-based factors that they felt hindered the optimal delivery of care for ARTIs.

Patients’ desire for antibiotics was the most common reason listed by physicians for possible antibiotic over-prescription. Other studies have shown that perceived patient demand for antibiotics is associated with antibiotic overuse and is a major barrier to providers’ adherence to ARTI guidelines [13, 15]. In this study, although only two patients indicated that they had specifically sought out antibiotics in the past, it is possible that physicians’ prescribing practices were colored by a few negative experiences or competition with the private sector. Continued physician education and reinforcement of ARTI guidelines, which can improve antibiotic prescribing practices for ARTIs, would be an important intervention in this OPD setting where physician awareness of antimicrobial resistance was low [6].

Diagnostic uncertainty and concern for bacterial superinfection was another reason cited by physicians for antibiotic over-prescription. Lower respiratory tract infections remain a leading cause of mortality among children in resource-limited settings, and may be a reason that physicians overprescribed antibiotics for ARTIs in this setting [16]. Other studies have documented that fear of missing a serious infection and diagnostic uncertainty can drive antibiotic use [17]. However, in our study, only two physicians suggested that increased access to diagnostic testing may be helpful, which may be due to the inability of current diagnostics to reliably distinguish between bacterial and viral infections [10]. Until the time that improved ARTI diagnostics are developed, physician training in managing diagnostic uncertainty will be important for reducing antibiotic over-prescription. Such training may cover the importance of adhering to guidelines, negotiating with patients regarding antibiotic use, and using teamwork with other physicians to ensure that a unified approach to antibiotic prescription is used [18].

Although asked open-ended questions regarding the etiology of their ARTIs, only a small minority of patients reported microbial causes for their ARTIs. The identification of illnesses as being either “hot” or “cold” has previously been described in Sri Lanka, and may be related to traditional beliefs regarding imbalances in the humoral system caused by diet and environment [19]. Patients expected some form of medication prescription from their OPD visit—not necessarily an antibiotic prescription-- and in fact received an average of three prescriptions per visit. Almost half of patients received a prescription for a steroid, and the rationale and downstream effects of this practice need to be explored further. Since many patients also endorsed multiple visits for the same illness, the implications of polypharmacy and drug interactions need to be explored in this outpatient setting where medical records are not maintained. In addition, the effectiveness of traditional therapies bears further investigation, as initial self-treatment with traditional therapies was common.

OPDs at all primary, secondary, and tertiary hospitals in Sri Lanka provide primary care to the population; patients are free to access care from the OPD of their choice [20]. The OPD system is heavily overburdened, as has been shown in other studies and was confirmed by this study [19]. In the past decades, the country has invested heavily in the public healthcare infrastructure and has achieved remarkable progress in universal healthcare coverage, with health indicators rivaling those of more developed nations [21]. However, the increase in access to care may have come with some trade-off in quality [19]. In the context of ARTI management in the OPD, time pressure and patient volume may adversely affect care delivery. Studies have shown that shorter visit times are associated with greater antibiotic prescriptions [11, 17]. In the busy OPD of this hospital in Sri Lanka, where typically more than 1000 patients are seen daily and individual patient visits tend to last less than 5 min, there is likely little time for clinicians to engage in rational decision-making. Although physicians had not described medication names or medication indications, possibly due to time pressure, most patients were satisfied with their visit and none expressed a desire to immediately seek care elsewhere [22]. However, physicians reported feeling overwhelmed by patient volume, especially since many patients were seeking care for minor complaints and had made multiple visits for the same illness. Public education to reduce care overutilization may be effective in this setting. Systematic changes such as reduction in patient volume through a referral system could be considered. Such changes may allow time for improved patient-provider communication, as prior studies have shown that patient satisfaction is associated with patients’ perception of care received and whether diagnoses and treatments are explained, rather than whether an antibiotic is prescribed [11].

A limitation to this study is the small number of physicians who were included, since we wished to restrict the study to the hospital where we had conducted our prior studies. The lack of involvement by all physicians in the OPD may have resulted in bias in the responses recorded. In addition, the physicians’ informational session was conducted after two physician interviews were completed, which may have resulted in differences in findings between the pre and post-session interviews. However, the informational session only covered basic findings from our prior studies; no recommendations regarding ARTI diagnosis or management were made. Thus, the probability of the latter interviews being influenced is low. Interviews were brief and in the context of a busy care environment, which may have impacted the responses given. Finally, social desirability bias on the part of either patients or physicians may have affected the responses. Strengths of this study include that the study was carried out in one of the largest public hospitals in Sri Lanka, functioning both as a primary and tertiary care center for the area. The study was carried out in the same OPD as our prior studies, and provided insight into effective interventions that could be carried out to reduce antibiotic over-prescription in the future. In addition, there are few qualitative studies of antibiotic prescribing for ARTIs in resource-limited settings, and to our knowledge, none simultaneously explore the views of both patients and physicians in these settings.

## Conclusions

In conclusion, antibiotic prescriptions for ARTIs were common at this public hospital in Sri Lanka, and were driven by physicians’ perception of patient demands, diagnostic uncertainty and fear of bacterial superinfection, as well as structural factors such as high patient volume and short visit times. Interventions that target education of prescribers, patients, and the public, in conjunction with systematic changes, are urgently needed to improve the rational and evidence-based prescription of antibiotics in resource-limited settings such as this one.

## Abbreviations

ARTI: Acute respiratory tract infection
OPD: Outpatient Department
ILI: Influenza-like illness

## References

[1] Donnelly JP, Baddley JW, Wang HE (2014). Antibiotic utilization for acute respiratory tract infections in U.S. emergency departments. Antimicrob Agents Chemother.

[2] Peltola V, Ruuskanen O (2008). Editorial commentary: respiratory viral infections in developing countries: common, severe, and unrecognized. Clin Infect Dis.

[3] Wang J, Wang P, Wang X, Zheng Y, Xiao Y (2014). Use and prescription of antibiotics in primary health care settings in China. JAMA Intern Med.

[4] Shapiro DJ, Hicks LA, Pavia AT, Hersh AL (2014). Antibiotic prescribing for adults in ambulatory care in the USA, 2007–09. J Antimicrob Chemother.

[5] Gill JM, Fleischut P, Haas S, Pellini B, Crawford A, Nash DB (2006). Use of antibiotics for adult upper respiratory infections in outpatient settings: a national ambulatory network study. Fam Med.

[6] Harris AM, Hicks LA, Qaseem A (2016). Appropriate antibiotic use for acute respiratory tract infection in adults: advice for high-value care from the american college of physicians and the centers for disease control and prevention. Ann Intern Med.

[7] Ozkaya E, Cambaz N, Coskun Y, Mete F, Geyik M, Samanci N (2009). The effect of rapid diagnostic testing for influenza on the reduction of antibiotic use in paediatric emergency department. Acta Paediatr.

[8] Tillekeratne L, Bodinayake C, Nagahawatte A, Vidanagama D, Devasiri V, Kodikara Arachchi W (2015). An under-recognized influenza epidemic identified by rapid influenza testing, southern Sri Lanka, 2013. Am J Trop Med Hyg.

[9] Tillekeratne LG, Bodinayake CK, Nagahawatte A, Vidanagama D, Devasiri V, Arachchi WK, et al. Use of Rapid Influenza Testing to Reduce Antibiotic Prescriptions Among Outpatients with Influenza-Like Illness in Southern Sri Lanka. Am J Trop Med Hyg. 2015;93(5):1031-7. 10.4269/ajtmh.15-0269 PMC4703267 26283748

[10] Zaas AK, Garner BH, Tsalik EL, Burke T, Woods CW, Ginsburg GS (2014). The current epidemiology and clinical decisions surrounding acute respiratory infections. Trends Mol Med.

[11] Linder JA, Singer DE, Stafford RS (2003). Association between antibiotic prescribing and visit duration in adults with upper respiratory tract infections. Clin Ther.

[12] World Health Organization. WHO Interim Global Epidemiological Surveillance Standards for Influenza. Geneva: World Health Organization; 2012. p. 1–61.

[13] Dempsey PP, Businger AC, Whaley LE, Gagne JJ, Linder JA (2014). Primary care clinicians inverted question mark perceptions about antibiotic prescribing for acute bronchitis: a qualitative study. BMC Fam Pract.

[14] Central Bank of Sri Lanka. Current Economic Indicators. 2010. http://www.cbsl.gov.lk/htm/english/_cei/er/e_1.asp. Accessed 10 Feb 2017.

[15] Lam TP, Lam KF (2003). What are the non-biomedical reasons which make family doctors over-prescribe antibiotics for upper respiratory tract infection in a mixed private/public Asian setting?. J Clin Pharm Ther.

[16] Lozano R, Naghavi M, Foreman K, Lim S, Shibuya K, Aboyans V (2012). Global and regional mortality from 235 causes of death for 20 age groups in 1990 and 2010: a systematic analysis for the global burden of disease study 2010. Lancet.

[17] Teixeira Rodrigues A, Roque F, Falcao A, Figueiras A, Herdeiro MT (2013). Understanding physician antibiotic prescribing behaviour: a systematic review of qualitative studies. Int J Antimicrob Agents.

[18] Danczak A, Lea A (2014). What do you do when you don’t know what to do? GP associates in training (AiT) and their experiences of uncertainty. Educ Prim Care.

[19] Russell S (2005). Treatment-seeking behaviour in urban Sri Lanka: trusting the state, trusting private providers. Soc Sci Med.

[20] Huntington D, Hort K. Public hospital governance in Asia and the Pacific. Geneva: World Health Organization. 2015. http://www.wpro.who.int/asia_pacific_observatory/country_comparative_studies/ccs_public_hospital_goverance.pdf?.

[21] Sri Lanka Ministry of Health and Indigenous Medicine. Colombo: Annual Health Bulletin. 2013.

[22] McGrath P, Henderson D, Tamargo J, Holewa HA (2012). Doctor-patient communication issues for international medical graduates: research findings from Australia. Educ Health (Abingdon).

